# HIN3/396: Experience of Creation of the National (Regional) Telematics Medical Corporate Network in Ukraine

**DOI:** 10.2196/jmir.1.suppl1.e34

**Published:** 1999-09-19

**Authors:** O Mayorov, V Ponomarenko, V Kalnish

**Affiliations:** ^1^Kharkiv Ukrainian Institute of Public HealthKharkivUkraine

**Keywords:** National Medical Networks, Teleconsulting Centres of EEG and ECG

## Abstract

**Introduction:**

Ukraine has an extensive experience on creating and maintaining National Medical Networks.

A variety of examples include:

- The National Register of the persons suffered from the Chernobyl disaster: Net of the Sanitary and Epidemiological Service of the Ministry of Health.
- The National Medical Network for monitoring oncology patients: this links Regional Oncology Dispensaries with Institute of Oncology.
- Ukraine has a membership to "EuroTransplant" and National Medical Network in this effort has linked Regional Centres with the National Informational Centre on Transplantation.
- Regional databases from Regional Venereologic Dispensaries, used for monitoring diseases, have been extended to include cases of AIDS.
- The Telemedical Cardiology Centre in Kharkiv, with trans-telephone ECG. All equipment, including portable ECG amplifiers and modems, is developed in the Ukraine.
- Teleconsulting Medical Centres, in Kiev, for the analysis of MRI images.
- The centre "Patholog", in which oncologists - cytologists carry out work.

The Ukrainian Association of Computer Medicine (UACM) has created (http: //www.uacm.cit-a.net) WWW- server , which has become a nucleus of the Corporate medical network permitting to exchange the information to 78 institutional members and over 900 individual members. The UACM has created affiliate Web - EHTO-UKRAINE server on national language (http://www.ehto-ukr.cit-ua.net). Ukraine took part in the "Telemedecine Medical Care Networks within the Baltic Region" programme. Also, Ukraine started the Ukrainian- American project on the monitoring of birth defects.

**Methods:**

The National Telematics Medical Corporate Network 'UkrMedNet' is under construction on the basis of existing the HealthNet and some other autonomous medical networks, with the newest of telecommunication technologies, used in Internet. This project also envisages the integration of all existing separate medical nets, universities and R&D institutes into one 'UkrMedNet', as well as the creation of a common informational space and its integration into the European one.

**Results:**

'UkrMedNet' has a three-level structure. The first level consists of the National and four Inter-Regional nodes, which have a similar structure and service 5 or 6 Districts. All Inter- Regional nodes are directly connected to: a) National node and b) one of Inter-Regional ones. Such connection layout ensures the "doubling" of the connections of the first level nodes. The second level is formed on the basis of the District Healthcare Departments. Nodes are connected to the nearest Inter- Regional node. They ensure connection to the net of medical organisations located in the district. The third level is Regional nodes and end users. They are connected to the District nodes.

**Discussion:**

The goal of the "UkrMed- Net' is to supply an infrastructure for all medical networks and Telemedicine Centres in Ukraine. It accelerates the integration of the country into the world informational space.

